# The Application of Nimotuzumab Combined With Definitive Chemoradiotherapy Toward the Treatment of Locally Advanced Cervical Esophageal Carcinoma: A Retrospective Study

**DOI:** 10.3389/fonc.2022.905422

**Published:** 2022-07-06

**Authors:** Jing Hu, Zhe Chen, Jiaming Lv, Zhen Zheng, Yanping Bei, Xue Chen, Lu Zheng, Wenjie Song, Yunbao Xu

**Affiliations:** ^1^ Department of Radiation Oncology, Ningbo Medical Center Lihuili Hospital, Ningbo, China; ^2^ Department of Thoracic Surgery, Ningbo Medical Center Lihuili Hospital, Ningbo, China; ^3^ Department of Radiotherapy and Chemotherapy, Hwamei Hospital, University of Chinese Academy of Sciences, Ningbo, China

**Keywords:** cervical esophageal carcinoma, nimotuzumab, intensity-modulated radiotherapy, chemoradiotherapy, C-reactive protein

## Abstract

**Objective:**

To evaluate the safety and effectiveness of nimotuzumab in combination with chemoradiotherapy for locally advanced cervical esophageal squamous cell carcinoma.

**Methods:**

Retrospective analysis was conducted from September 2012 to February 2017 among 50 locoregional-advanced cervical esophageal carcinoma (CEC) patients who received concurrent chemoradiotherapy (CRT) combined with or without nimotuzumab at Ningbo Medical Center Lihuili Hospital. Intensity-modulated radiotherapy (IMRT) was administrated on all patients. All patients were divided into two groups, of which 26 (Group A) received 200 mg (22 of 50) or 400 mg (4 of 50) of nimotuzumab per week with CRT and 24 (Group B) received definitive CRT.

**Results:**

The median follow-up time was 23 months. The median overall survival (OS) and progression-free survival (PFS) were 40.6 and 21.1 months for all, respectively. The 1-, 2-, and 3-year OS rates on the whole were 79.6%, 62.1%, and 47.8%. There was no statistical difference in overall response rate and disease control rate between the two groups. Patients treated with nimotuzumab (group A) had better PFS than the definitive CRT group (group B) (*P* < 0.05). However, the median OS was 41.4 months in group A and 32.4 months in group B, respectively (*P* = 0.517). Multivariate analysis showed that PFS among those with lower Eastern Cooperative Oncology Group (ECOG) score (HR = 5.11; *P* < 0.01), stage II (HR = 9.52; *P* < 0.01) and the application of nimotuzumab combined with CRT (HR = 0.16; *P* < 0.01) was much longer. Furthermore, ECOG, stage, C-reactive protein (CRP) baseline, and histological grade can also be used as independent predictors of OS. Grade >3 adverse reactions were not observed. The most common adverse event related to nimotuzumab was mild fever and the occurrence rate was 19% (5 of 26). The incidence of anemia was 65.4% in group A and 87.5% in group B (*P* < 0.05).

**Conclusions:**

For locoregional-advanced CEC, nimotuzumab combined with IMRT and concomitant chemotherapy was tolerated and effective. In addition, patients with a normal pretherapeutic serum CRP level (CRP < 10 mg/L) can achieve better OS.

## Introduction

Cervical esophageal carcinoma (CEC) is relatively rare and accounts for 2%–10% of all esophageal cancers (1). Squamous cell carcinoma is the major pathological type, which occupies 90% of CEC in China (2) and Northern America (3). The cervical esophagus, situated between the lower edge of the cricoid cartilage and the thoracic entrance (suprasternal notch), differs anatomically from other parts of the esophagus. Patients of CEC are often locally advanced with lymph node metastases, because it tends to infiltrate adjacent structures including the hypopharynx, trachea, and thyroid gland. Despite the fact that definitive surgery, particularly pharyngo-laryngo-esophagectomy, has been the initial treatment, the 3-year overall survival (OS) rate of CECs ranges from 24% to 45% (4–6). Moreover, definitive chemoradiotherapy (CRT) came to be a standard treatment modality for locally advanced CEC (LA-CEC) in consideration of the difficulty and complications of surgery and the potential of larynx preservation (7, 8).

In comparison to conventional and three-dimensional (3D) conformal radiotherapy (RT), several studies have shown that intensity-modulated RT (IMRT) provides great advantages in dose escalation, improved target volume coverage, and dose conformity along with reduced normal organ sparing (9–11). With the improvement of diagnosis and treatment techniques, the 5-year survival rate of CEC patients has elevated but still wandering about 30% (12, 13). Therefore, much more emphasis has been laid on the systemic treatment used within concurrent treatment regimens for CEC, such as the molecular targeted therapy. It has been demonstrated that the epidermal growth factor receptor (EGFR) pathway plays an important role in the growth, invasion and metastasis of tumor cells (14). It has also been reported that the overexpression rate of EGFR in esophageal squamous cell carcinoma (ESCC) is nearly 60%–70%, gene amplification 28% (15), and there is a close correlation between overexpression of EGFR and poor prognosis (16), which all indicates that EGFR may be one of the effective targets in the treatment of CEC.

Nimotuzumab, targeted to EGFR, is the recombinant humanized monoclonal antibody, which can strengthen chemotherapy sensibility, enhance RT efficacy, and inhibit tumor growth, metastasis, and local relapse (17). Furthermore, because of its highly human origination, rare adverse effect, and high-level safety, nimotuzumab has already gained good curative effect in many solid tumors (18). Some clinical trials indicated that nimotuzumab plus CRT were safe and provided statistically significant objective response in non-resectable esophageal cancer (19, 20). In addition, nimotuzumab combined with concurrent CRT in patients with unresectable locally advanced hypopharyngeal carcinoma had better short-term efficacy, OS, and progression-free survival (PFS) than patients without using nimotuzumab, with tolerable toxicity (21). Therefore, in this study, we retrospectively analyzed the treatment outcomes and side effects of nimotuzumab in patients with LA-CEC who were receiving concurrent CRT.

## Materials and Methods

### Patients

**Figure 1:**
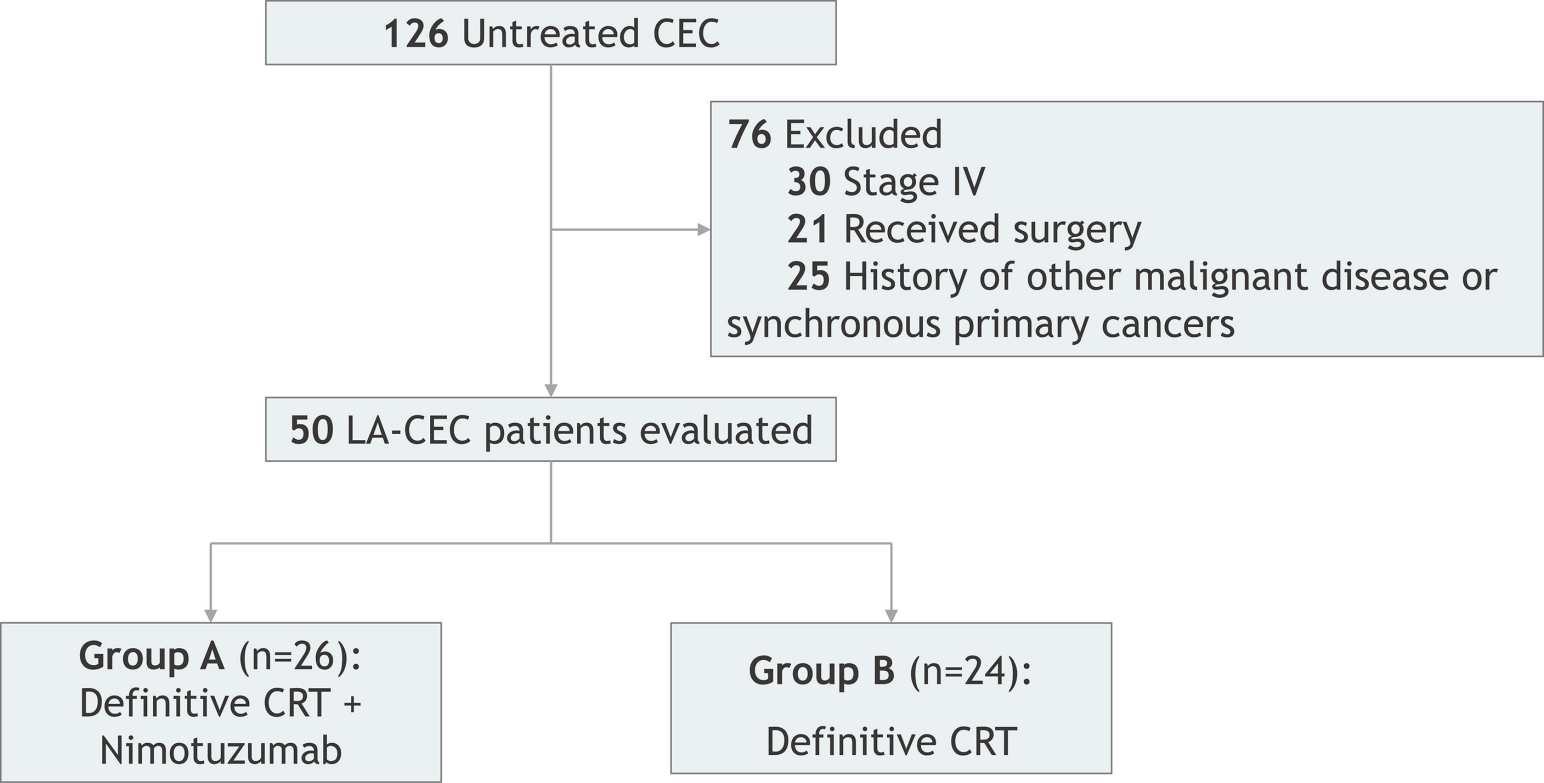
Enrollment diagram.

This was a retrospective study of CEC patients who underwent concurrent CRT combined with or without nimotuzumab at Ningbo Medical Center Lihuili Hospital from September 2012 to February 2017. A total of 50 patients were reviewed and met the following inclusion criteria: (1) aged between 18 and 75 years old; (2) diagnosed with cervical ESCC by histology or cytology; (3) with Eastern Cooperative Oncology Group (ECOG) ≥ 2; (4) with at least one measured lesion based on the response evaluation criteria in solid tumors (RECIST); (5) with no history of RT or receiving targeted therapy; (6) with no evidence of distant metastases; (7) with no CRT contraindication, no preperforation sign, no primary organs dysfunction, and blood routine test, biochemistry test, and heart and lung function are in basically normal condition. All patients were divided into two groups (**Figure 1**). Patients in group A (n = 26) received CRT with nimotuzumab, whereas patients in group B (n = 24) received definitive CRT (dCRT) without nimotuzumab. Both the patients and their family members have signed informed consent and this study was approved by the ethics committee of Ningbo Medical Center Lihuili Hospital.

### Treatment Method

#### Intensity-Modulated Radiotherapy

All patients received definitive IMRT with 6-MV photon beams. They were immobilized in the supine position by a fixed device and simulated by computed tomography (CT) scan. The range of CT scan was from the first cervical vertebra to under the diaphragm and can be increased by tumor condition. The scan slice thickness was 5 mm with vein injection of contrast agent. Target outline and treatment plans were optimized on the planning CT scan using the Pinnacle treatment planning system (version 9.8, Philips Medical Systems, Andover, MA). The gross tumor volume (GTV) included primary tumor identified by CT scan, barium esophagography and esophagoscope, and regional lymph nodes, which minor axis ≥1.0 cm or minor axis of tracheoesophageal groove lymph node ≥0.5 cm. Clinical target volume (CTV) was defined as the GTV plus a margin of 6 mm all round and of 3 cm in superior and inferior directions, as well as lymphatic involved-field irradiation or elective nodal irradiation, which included upper mediastinal areas and supraclavicular fossa with the upper margin at the caudal edge of cricoid cartilage, inferior margin at the sternal notch. A margin of 5 mm was extended three-dimensionally around CTV and GTV to form the planning target volume (PTV) and planning GTV (PGTV), respectively. The prescribed dose of 60–70 Gy was delivered to PGTV (2.0–2.14 Gy/fraction, five fractions per week) over 5–6 weeks. The prophylactic dose of 50–54 Gy was delivered to PTV (1.8–2.0 Gy/fraction, five fractions per week). The dose received by organs at risk (OAR) was under safety limitations.

#### Chemotherapy and Targeted Therapy

Platinum-based chemotherapy was administrated either induction chemotherapy or concurrent with RT, with or without adjuvant chemotherapy. The chemotherapy regimens consisted of cisplatin (75 mg/m^2^ d1-3) plus 5-fluorouracil (800 mg/m^2^/day, lasted 4 days)/capecitabine (850 mg/m^2^, bid d1-14), or cisplatin (75 mg/m^2^ d1-3) plus paclitaxel (135 mg/m^2^ d1) every 3 weeks. Cisplatin can be replaced by carboplatin (AUC = 5 d1) or nedaplatin (75 mg/m^2^ d1) because of side effects such as renal toxicity. Patients in group A received concurrent nimotuzumab (200 or 400 mg) intravenously once a week during RT.

#### Observation Index and Evaluation Criterion

Initial tumor response was evaluated by CT scan and/or esophagography at 1 and 4 months after the completion of treatment, respectively, according to RECIST version 1.1. Follow-up was conducted every 3 months for the first 2 years after treatment, every 6 months for the next 3 years, and annually afterward. Adverse events and toxicities during treatment were assessed and scored according to Radiation Therapy Oncology Group (RTOG) acute toxicity scoring system and Common Toxicity Criteria for Adverse Event (CTCAE version 4.0). The main observational end points were objective response rate (ORR), PFS, and OS. The responses included complete response, partial response, progression of disease, and stable disease. PFS was defined as the time gap from receiving therapy to disease progression, all-cause mortality, or follow-up terminal. OS was defined as the time gap from receiving therapy to all-cause mortality. All patients were followed by telephone and outpatient services.

### Statistical Analysis

The Statistical Package for Social Sciences software (version 19.0) was adopted to process data. Chi-square test or Fisher’s exact test is adopted to test the enumeration data. t-Test was adopted to test data with the quantitative data and expressed as *x ± s*. Locoregional control and survival data were estimated by the Kaplan–Meier method. The log-rank test was used in univariate analysis and the Cox proportional hazards model was used to determine any significant predictors of PFS and OS. *P* < 0.05 was considered as statistical significance. Variables with *P* < 0.05 in univariate analysis were selected to be included in multivariate analysis.

## Results

### Patients and Treatment Characteristics

During 2012–2017, 50 patients (42 male patients and 8 female patients) aged from 38 to 75 years old (median 62 years old) were enrolled in this research. Patient characteristics are shown in **Table 1**. All patients were pathologically confirmed as squamous cell carcinoma. Clinical stages were classified according to the seventh edition of the American Joint Committee on Center (AJCC) staging system, including 19 stage II cases (38%) and 31 stage III cases (62%). Imaging examinations showed that the median length of lesions was 4.1 cm (2–8 cm), and the median width was 2.5 cm (0.6–4.8 cm). In addition, tumors extended to the hypopharynx in 10 patients.

**Table 1:** Characteristics of the patients at baseline.

Characteristic	All cases (n = 50) (%)	Group A (n = 26) (%)	Group B (n = 24) (%)	*P*
**Gender**				0.155
Male	42 (84)	20 (76.9)	22 (91.7)	
Female	8 (16)	6 (23.1)	2 (8.3)	
**Age(years)**				0.665
≥62	26 (52)	13 (50)	13 (54.2)	
< 62	24 (48)	13 (50)	11 (45.8)	
**ECOG**				0.516
0	16 (32)	10 (38.5)	6 (25)	
1	24 (48)	12 (46.2)	12 (50)	
2	10 (20)	4 (15.4)	6 (25)	
**Histological grade**				0.614
G1	12 (24)	7 (26.9)	5 (20.8)	
G2-3	38 (76)	19 (73.1)	19 (79.2)	
**Tumor length (cm)**				0.423
≥4	18 (36)	8 (30.8)	10 (41.7)	
< 4	32 (64)	18 (69.2)	14 (58.3)	
**AJCC stage**				0.944
II	19 (38)	10 (38.5)	9 (37.5)	
III	31( 62)	16 (61.5)	15 (62.5)	
**Induction chemotherapy**				0.887
Yes	10 (20)	5 (19.2)	5 (20.8)	
No	40 (80)	21 (80.8)	19 (79.2)	
**Adjuvant chemotherapy**				0.308
Yes	16 (32)	10 (38.5)	6 (25)	
No	34 (68)	16 (61.5)	18 (75)	
**CRP (mg/L)**				0.598
≥10	21 42)	10 (38.5)	11 (45.8)	
<10	29 (58)	16 (61.5)	13 (54.2)	

Ten patients (20%) received induction chemotherapy, and 16 (32%) received adjuvant chemotherapy. Moreover, the combined regimen that included fluorouracil was administrated in 34 patients, whereas the paclitaxel regimen was adopted in 16 patients. All patients were treated with IMRT. The median radiation dose was 66 Gy for PGTV and 52 Gy for PTV. Among 26 patients who received concurrent CRT with nimotuzumab, 22 patients (84.6%) received 200 mg of nimotuzumab per week, 4 (15.4%) received 400 mg per week, and 12 (46.2%) received a total dose of greater than 1,200 mg. The median cycle of nimotuzumab was 6 weeks (4–7 weeks).

### Toxicity

Toxicity was recorded according to Nation Cancer Institute CTCAE v4.0. The most common adverse reactions include radiation esophagitis (100%), leucopenia (88%), and anemia (72%). The incidence of anemia was statistically different between the two groups (65.4% vs. 87.5%, *P* < 0.05). Grade 3 of radiation pneumonitis and leucopenia were observed in 2 and 10 patients respectively, but there was no difference in incidence between the two groups. No patients developed tracheoesophageal fistula during or after RT. The most common adverse event related to nimotuzumab was mild fever and the occurrence rate was 10% (5 of 50). Other adverse events including polyserositis, diarrhea, intestinal fungal infection, and electrolyte disturbance were sporadic. None of grade 4 toxicity was occurred in all patients. Patients from both groups were generally well tolerated by treatment. Specific adverse events were shown in **Table 2**.

**Table 2:** Treatment-related adverse events in 50 patients.

Adverse events	Total (n = 50)				Group A (n = 26)		Group B (n = 24)		*P*
	^a^G1 (%)	G2 (%)	G3 (%)	G4 (%)	G1-2 (%)	G3-4 (%)	G1-2 (%)	G3-4 (%)	
**Radiation-related**									
Esophagitis	22 (44)	23 (46)	5 (10)	0	24 (92.3)	2 (7.7)	21 (87.5)	3 (12.5)	0.661
Pneumonitis/bronchitis	11 (22)	4 (8)	2 (4)	0	5 (19.2)	1 (3.9)	10 (41.7)	1 (4.2)	0.168
Skin reaction in radiation fields	7 (14)	3 (6)	0	0	5 (19.2)	0 (0)	5 (20.8)	0 (0)	1.000
**Chemotherapy-related**									
Nausea/vomiting	8 (16)	4 (8)	0	0	5 (19.2)	0	7 (29.2)	0	0.514
Anorexia	2 (4)	0	0	0	1 (3.8)	0	1 (4.2)	0	1.000
Alopecia	18 (36)	6 (12)	0	0	14 (53.8)	0	10 (41.7)	0	0.564
Leucopenia	21 (42)	13 (26)	10 (20)	0	19 (73.1)	6 (25)	15 (62.5)	4 (16.7)	0.214
Thrombocytopenia	7 (14)	4 (8)	2 (4)	0	6 (23.1)	0	5 (20.8)	2 (8.3)	0.445
Anemia	19 (38)	17 (34)	2 (4)	0	16 (61.5)	1 (3.8)	20 (83.3)	1 (4.2)	**0.048**
Fatigue	14 (28)	9 (18)	0	0	10 (38.5)	0	13 (54.2)	0	0.395
**Nimotuzumab-related**									
Fever/Chill	4 (8)	1 (2)	0	0	4 (15.4)	1 (3.8)	–	–	
Skin rash	1 (2)	0	0	0	1 (3.8)	0	–	–	

a G means Grade.

### Therapeutic Effectiveness

All patients have finished short-term curative effect evaluation (**Table 3**). The assessment was performed at 1 and 4 months after finishing concurrent CRT. The first evaluation result showed that an overall ORR was 64% (32 of 50), compared with 73% (19 of 26) for group A and 54% (13 of 24) for group B. The disease control rate (DCR) for all cases was 98% (49 of 50), 96% (25 of 26) for group A and 100% (24 of 24) for group B. The second evaluation showed that the ORR was 78% with DCR and was 90% for all patients. There were no significant differences in ORR and DCR rates between the two groups at two different assessment times (*P* > 0.05).

**Table 3:** Response to treatment.

Response	Total		Group A		Group B	
	1st^a^	2nd^b^	1st^a^	2nd^b^	1st^a^	2nd^b^
**CR**	2	5	2	2	0	3
**PR**	30	34	17	20	13	14
**SD**	17	6	6	2	11	4
**PD**	1	5	1	2	0	3

a First evaluation: 1 month after the radiation.

b Second evaluation: 4 months after the radiation.

CR, complete response; PR, partial response; PD, progression of disease; SD, stable disease.

### Survival Analysis

**Figure 2:**
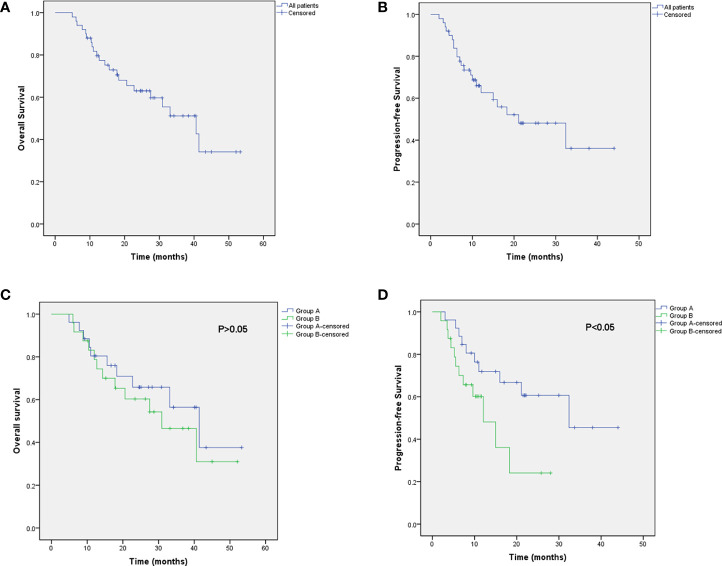
Kaplan–Meier curve of OS **(A)** and PFS **(B)** for all patients. Kaplan–Meier curve of progression-free survival for all patients. Kaplan–Meier analysis of OS **(C)** and PFS **(D)** in group A and group B.

All patients were followed up until death or the time of the last follow-up evaluation. The terminal follow-up date was November 2020, and the period ranged from 5 to 53 months (the median follow-up period: 23 months). The median OS and PFS were 40.6 and 21.1 months for all, respectively. The median OS and PFS in group A and group B were 41.4 vs. 30.9 m (*P* = 0.517), and 32.4 vs. 12.1 m (*P* = 0.048), respectively. In addition, the 1-, 2-, and 3-year OS rates of the whole were 79.6%, 62.1%, and 47.8%. The 1- and 3-year OS rates were 79.4% and 49.8% in group A, and 78.8% and 38.4% in group B. The 1-year PFS rate was 70.9% in group A, compared with 48.1% in group B (**Figure 2**).

The underlying correlation analysis toward OS and PFS was performed. Log-rank univariate analysis showed that the OS was associated with ECOG score, histological grade, tumor length, stage, and C-reactive protein (CRP) baseline before treatment, whereas PFS was related to ECOG, histological grade, stage, adjuvant chemotherapy and CRP baseline, and the application of nimotuzumab (**Table 4**). Further Cox multivariate analysis was presented in **Table 5**. As a result, ECOG, histological grade, stage, and CRP baseline were independent prognostic factors for OS. Compared to patients with lower ECOG, stage II, and the combined application of nimotuzumab, the PFS among those with higher ECOG, stage III, and concurrent CRT was much shorter.

**Table 4:** Univariate analyses of clinical parameters of OS and PFS in overall patients.

	Overall survival		Progression-free survival	
	Median (m)	*P*	Median (m)	*P*
**Gender**				
Male	34.4	0.427	25.2	0.729
Female	23.0		14.9	
**Age(years)**				
≥62	27.0	0.251	16.3	0.365
< 62	36.0		27.7	
**ECOG**				
0	46.7	**<0.01**	37.3	**<0.01**
1	28.5		20.1	
2	11.6		6.0	
**Histological grade**				
G1	40.8	**<0.01**	35.2	**<0.01**
G2-3	28.9		20.4	
**Tumor length (cm)**				
≥4	18.5	**<0.05**	13.0	0.052
<4	37.3		27.4	
**AJCC stage**				
II	43.4	**<0.01**	31.5	**<0.01**
III	18.6		13.1	
**Induction chemotherapy**				
Yes	31.1	0.130	21.2	0.148
No	31.3		23.1	
**Adjuvant chemotherapy**				
Yes	39.8	0.074	31.1	**<0.05**
No	30.6		17.8	
**CRP (mg/L)**				
<10	40.9	**<0.01**	31.7	**<0.01**
≥10	20.7		12.5	
**Nimotuzumab**				
Yes	41.1	0.517	32.4	**<0.05**
No	30.9		12.1	
**Nimotuzumab per week**				
200 mg/week	30.6	0.669	24.9	0.572
400 mg/week	37.4		31.6	
**Total Nimotuzumab**				
<1200 mg	28.7	0.283	20.2	0.209
≥1,200 mg	37.7		31.8	

**Table 5:** Multivariate analyses of clinical parameters of OS and PFS in overall patients.

Factor	OS		PFS	
	HR (95%CI)	*P*	HR (95%CI)	*P*
**ECOG**				
0/1/2	6.08 (2.32~15.90)	**<0.01**	5.11 (2.29~11.38)	**<0.01**
**Histological grade**				
G2~3/G1	14.61 (1.18~180.88)	0.037		0.138
**Stage**				
II/III	38.30 (5.60~262.01)	**<0.01**	9.52 (2.72~33.31)	**<0.01**
**Nimotuzumab**				
Yes/No	–	0.839	0.16 (0.05~0.50)	**<0.01**
**CRP**				
<10/≥10	2.79 (1.00~7.73)	**0.049**		0.307

## Discussion

Considering the low incidence and dismal prognosis of CEC, research on treatment is relatively restricted. The efficacy and safety of nimotuzumab combined with IMRT and concurrent chemotherapy in LA-CEC have been investigated for the first time in this trial. The malignant potential of CEC is not higher than that of other regions of esophageal carcinoma, according to Saeki et al. (22). They also discovered that women were more prevalent in the CEC group than in the thoracic or abdominal esophageal cancer groups, and that females and males had biological disparities. The majority of CECs are locally progressed at the time of initial diagnosis, with 55% of stage III or IV and 27% of stage II (12, 23, 24).

So far, the optimal method of treating CEC is still controversial. Historically, surgery has been a radical procedure, but it has not been widely accepted due to its negative impact on patients’ quality of life, such as dysfunction of speech and swallowing. RT plays an irreplaceable role in local therapy of esophageal carcinoma, whereas chemotherapy can control disease progression systemically. Therefore, noninvasive therapeutic strategies such as RT with or without chemotherapy are gradually introduced. Takebayashi et al. compared dCRT and curative resection as initial treatment in patients with resectable CEC and found that the 5-year OS rates were 51.4% and 60.6%, respectively (*P* = 0.89), implying that these two strategies had comparable survival outcomes (25). They also proposed that some patients with residual tumor in dCRT group who underwent salvage surgery achieved a similar 5-year survival rate as those in the surgery group. Furthermore, several studies have looked into the advantages of IMRT in CEC (26–28). Compared with 2D-RT and 3D conformal RT, IMRT combined with concurrent chemotherapy had reduced incidence of late toxicity and accomplished better OS rate (9, 29). However, there was no big breakthrough in OS for LA-CEC.

As the cervical esophagus is located at the junction of the hypopharynx and thoracic esophagus, its treatment schedule is sometimes referred to squamous cell carcinoma of the head and neck. In unresectable locally advanced hypopharyngeal cancer patients, Tian et al. observed that utilizing nimotuzumab in combination with induction chemotherapy and concurrent CRT resulted in better short-term efficacy, as well as improved OS and PFS, than patients who did not receive nimotuzumab (21). The EGFR pathway has been involved in the pathogenesis and development of epithelioma, including esophageal cancer. It has been indicated that overexpression of EGFR is quite common in ESCC, which has profound correlations with prognosis (15, 16, 30). However, the multicenter phase II/III SCOPEI trail and the randomized phase III RTOG 0436 trail found no improvement in OS when esophageal cancer patients received dCRT with or without cetuximab, probably due to its toxicity (31, 32), whereas some scholars have noted a difference in DCRs of 75% and 57%, respectively, and median OS of 9.5 and 5.5 months for standard chemotherapy with or without cetuximab in metastatic ESCC (33). Another anti-EGFR monoclonal antibody, nimotuzumab, has shown promising results in a variety of solid tumors, such as head and neck neoplasm, non–small cell lung cancer, neuroglioma, and pancreatic cancer (34–37). Various studies have been focused on the curative effect of nimotuzumab combined with RT or CRT in esophageal carcinoma, and the majority of the results appeared to be favorable (19, 38). In a prospective phase II clinical trial conducted in China, 56 patients with unresectable locally advanced or metastatic ESCC were enrolled (39). With an ORR of 51.8% and the median OS of 20.2 months, combining nimotuzumab with paclitaxel and cisplatin resulted in a stronger antitumor effect. Among 29 patients with local regional ESCC, the ORR was 41.4% and median OS had not yet been reached. A retrospective study conducted by Ma et al. analyzed 66 ESCC patients who received nimotuzumab in combination with RT or CRT (40). The result turned out that the median OS was 26.0 months and the median PFS was 16.7 months. Moreover, the 2-year OS, PFS, and local control rates were 54%, 37%, and 80%, respectively. Another retrospective study evaluated the safety and efficacy of nimotuzumab in combination with chemotherapy in patients with locally advanced or metastatic esophageal cancer (41). It revealed promising clinical outcomes with DCR of 81% and median OS of 18 months. In these investigations, nimotuzumab had less toxicity than cetuximab, especially in the case of skin rashes. There were no toxic reactions of grades 3–4 either.

Thus, it is necessary to investigate whether nimotuzumab in combination with CRT has the same efficacy in cervical as it does in thoracic and abdominal esophageal cancer. In this trial, patients in nimotuzumab group had equivalent ORR and DCR in early efficacy assessments in comparison with the CRT group. Although there was no significant difference in median OS between the two groups, the median PFS in the combination group was superior to that in the CRT group (*P* < 0.05). Nimotuzumab combined with concurrent CRT manifested good tolerance, with no therapy-related death. Mild fever was the most common adverse event associated with nimotuzumab with an occurrence rate of 10% (5 of 50). Only 1 patient out of 50 had a slight rash in the back, which disappeared spontaneously. Additionally, acne-like rash, the most common adverse event caused by EGFR-targeted therapies (42), was not observed and the underlying reason might be related to the relatively lower affinity and less binding avidity of nimotuzumab (18). Crombet and his colleagues (43) created a mathematical model that predicted the greatest difference between the area under the curve (AUC) of tumors (high tumor uptake) and normal tissues (low uptake) when the antibody had a moderate affinity (10^−8^ to 10^−9^ M), and the affinity of nimotuzumab was within this optimal range. In contrast to cetuximab, nimotuzumab can selectively bind to cells expressing moderate to high EGFR levels. In normal tissues with low EGFR expression, nimotuzumab binding interaction is transient, thus avoiding severe dose-limiting toxicity. However, both antibodies revealed similar tumor growth inhibiting in tumor tissue with high EGFR expression (44). We also discovered that the application of nimotuzumab might reduce the incidence of anemia, which has not been reported in other previous studies. Multivariate analysis revealed that CEC patients with a higher ECOG score, lower histological grade, and earlier stage had a better OS, which was in accordance with the prior research (45). Interestingly, our research also found that patients with normal CRP baseline (CRP ≤ 10 mg/L) can achieve better OS than those with CRP >10. According to certain studies, serum CRP concentration before treatment can be a predicted factor of curative response toward neoadjuvant CRT among local advanced esophageal carcinoma patients (46). Patients with normal CRP levels had significant survival advantage over those with high levels. Meta-analysis also demonstrated that esophageal carcinoma patients with a medium or high level of CRP had much worse OS than those of normal CRP (47); thus, we speculate that patients with normal pretherapeutic CRP levels will benefit more from the nimotuzumab treatment.

It is unfortunate that this research did not find the correlations between curative effect and the dose and period of nimotuzumab. Studies of Xu et al. (48) and Wang et al. (49) showed that it can benefit more if one received >6 cycles and >200 mg per week. Another phase I research about step-up dosage of nimotuzumab combined with CRT showed that 400 mg of nimotuzumab per week with RT was a safe and tolerable dosage for esophageal patients with advanced stage and the adverse event did not increase significantly (50). Therefore, larger samples are needed to be included to demonstrate whether the increase in dosage is proportional to the curative effect.

## Conclusion

In summary, nimotuzumab combined with CRT is safe and tolerable with favorable survival outcomes in LA-CEC. In addition, patients with a normal serum CRP level (CRP <10 mg/L) before treatment can have better OS.

## Data Availability Statement

The original contributions presented in the study are included in the article/supplementary material. Further inquiries can be directed to the corresponding author.

## Ethics Statement

The studies involving human participants were reviewed and approved by the Ethics Committee of Ningbo Medical Center Lihuili Hospital. The patients/participants provided their written informed consent to participate in this study.

## Author Contributions

Conception and design: JH and YX; Provision of study materials or patients: ZC, YB, LZ, XC, and WS; Collection and assembly of data: JL, ZC, and ZZ; Data analysis and interpretation: YX, JL, and ZZ; Manuscript writing: JH; Review and editing: XC, YB, LZ, and YX; Final approval of manuscript: All authors.

## Funding

This study is supported by Natural Science Foundation of Ningbo (Grant number: 2021J293).

## Conflict of Interest

The authors declare that the research was conducted in the absence of any commercial or financial relationships that could be construed as a potential conflict of interest.

## Publisher’s Note

All claims expressed in this article are solely those of the authors and do not necessarily represent those of their affiliated organizations, or those of the publisher, the editors and the reviewers. Any product that may be evaluated in this article, or claim that may be made by its manufacturer, is not guaranteed or endorsed by the publisher.

## References

[1] Lee DJ Harris A Gillette A Munoz L Kashima H. Carcinoma of the Cervical Esophagus: Diagnosis, Management, and Results. South Med J (1984) 77(11):1365–7. doi: 10.1097/00007611-198411000-00004 6494955

[2] Chen W Zheng R Baade PD Zhang S Zeng H Bray F. Cancer Statistics in China, 2015. CA Cancer J Clin (2016) 66(2):115–32. doi: 10.3322/caac.21338 26808342

[3] Davies L Welch HG. Epidemiology of Head and Neck Cancer in the United States. Otolaryngol Head Neck Surg (2006) 135(3):451–7. doi: 10.1016/j.otohns.2006.01.029 16949981

[4] Triboulet JP Mariette C Chevalier D Amrouni H. Surgical Management of Carcinoma of the Hypopharynx and Cervical Esophagus: Analysis of 209 Cases. Arch Surg (2001) 136(10):1164–70. doi: 10.1001/archsurg.136.10.1164 11585510

[5] Miyata H Yamasaki M Takahashi T Kurokawa Y Nakajima K Takiguchi S. Larynx-Preserving Limited Resection and Free Jejunal Graft for Carcinoma of the Cervical Esophagus. World J Surg (2013) 37(3):551–7. doi: 10.1007/s00268-012-1875-7 23224075

[6] Sun F Li X Lei D Jin T Liu D Zhao H. Surgical Management of Cervical Esophageal Carcinoma With Larynx Preservation and Reconstruction. Int J Clin Exp Med (2014) 7(9):2771–8. PMC4211788 25356138

[7] Ajani JA D'Amico TA Almhanna K Bentrem DJ Besh S Chao J. Esophageal and Esophagogastric Junction Cancers, Version 1.2015. J Natl Compr Canc Netw (2015) 13(2):194–227. doi: 10.6004/jnccn.2015.0028 25691612

[8] Lordick F Mariette C Haustermans K Obermannova R Arnold D Committee EG. Oesophageal Cancer: Esmo Clinical Practice Guidelines for Diagnosis, Treatment and Follow-Up. Ann Oncol (2016) 27(suppl 5):v50–v7. doi: 10.1093/annonc/mdw329 27664261

[9] Cao C Luo J Gao L Xu G Yi J Huang X. Definitive Intensity-Modulated Radiotherapy Compared With Definitive Conventional Radiotherapy in Cervical Oesophageal Squamous Cell Carcinoma. Radiol Med (2015) 120(7):603–10. doi: 10.1007/s11547-015-0510-8 25644251

[10] Fenkell L Kaminsky I Breen S Huang S Van Prooijen M Ringash J. Dosimetric Comparison of Imrt Vs. 3d Conformal Radiotherapy in the Treatment of Cancer of the Cervical Esophagus. Radiother Oncol (2008) 89(3):287–91. doi: 10.1016/j.radonc.2008.08.008 18789828

[11] Lu JY Cheung ML Huang BT Wu LL Xie WJ Chen ZJ. Improving Target Coverage and Organ-At-Risk Sparing in Intensity-Modulated Radiotherapy for Cervical Oesophageal Cancer Using a Simple Optimisation Method. PLoS One (2015) 10(3):e0121679. doi: 10.1371/journal.pone.0121679 25768733 PMC4358965

[12] Huang SH Lockwood G Brierley J Cummings B Kim J Wong R. Effect of Concurrent High-Dose Cisplatin Chemotherapy and Conformal Radiotherapy on Cervical Esophageal Cancer Survival. Int J Radiat Oncol Biol Phys (2008) 71(3):735–40. doi: 10.1016/j.ijrobp.2007.10.022 18164844

[13] Gkika E Gauler T Eberhardt W Stahl M Stuschke M Pottgen C. Long-Term Results of Definitive Radiochemotherapy in Locally Advanced Cancers of the Cervical Esophagus. Dis Esophagus (2014) 27(7):678–84. doi: 10.1111/dote.12146 24147973

[14] Modjtahedi H Essapen S. Epidermal Growth Factor Receptor Inhibitors in Cancer Treatment: Advances, Challenges and Opportunities. Anticancer Drugs (2009) 20(10):851–5. doi: 10.1097/CAD.0b013e3283330590 19826350

[15] Ayyappan S Prabhakar D Sharma N. Epidermal Growth Factor Receptor (Egfr)-Targeted Therapies in Esophagogastric Cancer. Anticancer Res (2013) 33(10):4139–55. 24122977

[16] Jiang D Li X Wang H Shi Y Xu C Lu S. The Prognostic Value of Egfr Overexpression and Amplification in Esophageal Squamous Cell Carcinoma. BMC Cancer (2015) 15:377. doi: 10.1186/s12885-015-1393-8 25953424 PMC4437683

[17] Crombet-Ramos T Rak J Perez R Viloria-Petit A. Antiproliferative, Antiangiogenic and Proapoptotic Activity of H-R3: A Humanized Anti-Egfr Antibody. Int J Cancer (2002) 101(6):567–75. doi: 10.1002/ijc.10647 12237899

[18] Ramakrishnan MS Eswaraiah A Crombet T Piedra P Saurez G Iyer H. Nimotuzumab, a Promising Therapeutic Monoclonal for Treatment of Tumors of Epithelial Origin. MAbs (2009) 1(1):41–8. doi: 10.4161/mabs.1.1.7509 PMC2715181 20046573

[19] Ramos-Suzarte M Lorenzo-Luaces P Lazo NG Perez ML Soriano JL Gonzalez CE. Treatment of Malignant, Non-Resectable, Epithelial Origin Esophageal Tumours With the Humanized Anti-Epidermal Growth Factor Antibody Nimotuzumab Combined With Radiation Therapy and Chemotherapy. Cancer Biol Ther (2012) 13(8):600–5. doi: 10.4161/cbt.19849 22555809

[20] Kato K Ura T Koizumi W Iwasa S Katada C Azuma M. Nimotuzumab Combined With Concurrent Chemoradiotherapy in Japanese Patients With Esophageal Cancer: A Phase I Study. Cancer Sci (2018) 109(3):785–93. doi: 10.1111/cas.13481 PMC5834813 29285832

[21] Tian X Xuan Y Wu R Gao S. Nimotuzumab Combined With Induction Chemotherapy and Concurrent Chemoradiotherapy in Unresectable Locally Advanced Hypopharyngeal Carcinoma: A Single Institution Experience in China. Cancer Manag Res (2020) 12:3323–9. doi: 10.2147/CMAR.S248392 PMC7227783 32494195

[22] Saeki H Tsutsumi S Yukaya T Tajiri H Tsutsumi R Nishimura S. Clinicopathological Features of Cervical Esophageal Cancer: Retrospective Analysis of 63 Consecutive Patients Who Underwent Surgical Resection. Ann Surg (2017) 265(1):130–6. doi: 10.1097/SLA.0000000000001599 28009737

[23] Grass GD Cooper SL Armeson K Garrett-Mayer E Sharma A. Cervical Esophageal Cancer: A Population-Based Study. Head Neck (2015) 37(6):808–14. doi: 10.1002/hed.23678 24616217

[24] Yamada K Murakami M Okamoto Y Okuno Y Nakajima T Kusumi F. Treatment Results of Radiotherapy for Carcinoma of the Cervical Esophagus. Acta Oncol (2006) 45(8):1120–5. doi: 10.1080/02841860600609768 17118849

[25] Takebayashi K Tsubosa Y Matsuda S Kawamorita K Niihara M Tsushima T. Comparison of Curative Surgery and Definitive Chemoradiotherapy as Initial Treatment for Patients With Cervical Esophageal Cancer. Dis Esophagus (2017) 30(2):1–5. doi: 10.1111/dote.12502 27859977

[26] Zhang P Xi M Zhao L Qiu B Liu H Hu YH. Clinical Efficacy and Failure Pattern in Patients With Cervical Esophageal Cancer Treated With Definitive Chemoradiotherapy. Radiother Oncol (2015) 116(2):257–61. doi: 10.1016/j.radonc.2015.07.011 26233590

[27] Li HX Liu J Cheng Y Liu MN Fang WT Lv CX. Concurrent chemoradiotherapy for cervical esophageal squamous cell carcinoma: treatment results from a prospective observational study. Dis Esophagus (2018) 31(5):1–6. doi: 10.1093/dote/dox144 29294022

[28] Zhang J Zhang W Zhang B Qian D Li X Zhang H. Clinical Results of Intensity-Modulated Radiotherapy for 250 Patients With Cervical and Upper Thoracic Esophageal Carcinoma. Cancer Manag Res (2019) 11:8285–94. doi: 10.2147/CMAR.S203575 PMC6748035 31571986

[29] Ito M Kodaira T Tachibana H Tomita N Makita C Koide Y. Clinical Results of Definitive Chemoradiotherapy for Cervical Esophageal Cancer: Comparison of Failure Pattern and Toxicities Between Intensity-Modulated Radiotherapy and 3-Dimensional Conformal Radiotherapy. Head Neck (2017) 39(12):2406–15. doi: 10.1002/hed.24909 28960561

[30] Lin G Sun XJ Han QB Wang Z Xu YP Gu JL. Epidermal Growth Factor Receptor Protein Overexpression and Gene Amplification Are Associated With Aggressive Biological Behaviors of Esophageal Squamous Cell Carcinoma. Oncol Lett (2015) 10(2):901–6. doi: 10.3892/ol.2015.3277 PMC4509062 26622592

[31] Crosby T Hurt CN Falk S Gollins S Mukherjee S Staffurth J. Chemoradiotherapy With or Without Cetuximab in Patients With Oesophageal Cancer (Scope1): A Multicentre, Phase 2/3 Randomised Trial. Lancet Oncol (2013) 14(7):627–37. doi: 10.1016/S1470-2045(13)70136-0 23623280

[32] Suntharalingam M Winter K Ilson D Dicker AP Kachnic L Konski A. Effect of the Addition of Cetuximab to Paclitaxel, Cisplatin, and Radiation Therapy for Patients With Esophageal Cancer: The Nrg Oncology Rtog 0436 Phase 3 Randomized Clinical Trial. JAMA Oncol (2017) 3(11):1520–8. doi: 10.1001/jamaoncol.2017.1598 PMC5710193 28687830

[33] Lorenzen S Schuster T Porschen R Al-Batran SE Hofheinz R Thuss-Patience P. Cetuximab Plus Cisplatin-5-Fluorouracil Versus Cisplatin-5-Fluorouracil Alone in First-Line Metastatic Squamous Cell Carcinoma of the Esophagus: A Randomized Phase Ii Study of the Arbeitsgemeinschaft Internistische Onkologie. Ann Oncol (2009) 20(10):1667–73. doi: 10.1093/annonc/mdp069 19549707

[34] Babu KG Prabhash K Vaid AK Sirohi B Diwakar RB Rao R. Nimotuzumab Plus Chemotherapy Versus Chemotherapy Alone in Advanced Non-Small-Cell Lung Cancer: A Multicenter, Randomized, Open-Label Phase Ii Study. Onco Targets Ther (2014) 7:1051–60. doi: 10.2147/OTT.S63168 PMC4063861 24966687

[35] Reddy BK Lokesh V Vidyasagar MS Shenoy K Babu KG Shenoy A. Nimotuzumab Provides Survival Benefit to Patients With Inoperable Advanced Squamous Cell Carcinoma of the Head and Neck: A Randomized, Open-Label, Phase Iib, 5-Year Study in Indian Patients. Oral Oncol (2014) 50(5):498–505. doi: 10.1016/j.oraloncology.2013.11.008 24613543

[36] Solomon MT Selva JC Figueredo J Vaquer J Toledo C Quintanal N. Radiotherapy Plus Nimotuzumab or Placebo in the Treatment of High Grade Glioma Patients: Results From a Randomized, Double Blind Trial. BMC Cancer (2013) 13:299. doi: 10.1186/1471-2407-13-299 23782513 PMC3691625

[37] Schultheis B Reuter D Ebert MP Siveke J Kerkhoff A Berdel WE. Gemcitabine Combined With the Monoclonal Antibody Nimotuzumab Is an Active First-Line Regimen in Kras Wildtype Patients With Locally Advanced or Metastatic Pancreatic Cancer: A Multicenter, Randomized Phase Iib Study. Ann Oncol (2017) 28(10):2429–35. doi: 10.1093/annonc/mdx343 28961832

[38] Liang J E M Wu G Zhao L Li X Xiu X. Nimotuzumab Combined With Radiotherapy for Esophageal Cancer: Preliminary Study of a Phase II Clinical Trial. Oncol Targets Ther (2013) 6:1589–96. doi: 10.2147/OTT.S50945 PMC3825695 24235844

[39] Lu M Wang X Shen L Jia J Gong J Li J. Nimotuzumab Plus Paclitaxel and Cisplatin as the First Line Treatment for Advanced Esophageal Squamous Cell Cancer: A Single Centre Prospective Phase Ii Trial. Cancer Sci (2016) 107(4):486–90. doi: 10.1111/cas.12894 PMC4832869 26797530

[40] Ma NY Cai XW Fu XL Li Y Zhou XY Wu XH. Safety and Efficacy of Nimotuzumab in Combination With Radiotherapy for Patients With Squamous Cell Carcinoma of the Esophagus. Int J Clin Oncol (2014) 19(2):297–302. doi: 10.1007/s10147-013-0564-3 23690261

[41] Han X Lu N Pan Y Xu J. Nimotuzumab Combined With Chemotherapy Is a Promising Treatment for Locally Advanced and Metastatic Esophageal Cancer. Med Sci Monit (2017) 23:412–8. doi: 10.12659/msm.902645 PMC5286920 28115730

[42] Ocvirk J Heeger S McCloud P Hofheinz RD. A Review of the Treatment Options for Skin Rash Induced by Egfr-Targeted Therapies: Evidence From Randomized Clinical Trials and a Meta-Analysis. Radiol Oncol (2013) 47(2):166–75. doi: 10.2478/raon-2013-0014 PMC3691090 23801914

[43] Crombet T Osorio M Cruz T Roca C del Castillo R Mon R. Use of the Humanized Anti-Epidermal Growth Factor Receptor Monoclonal Antibody H-R3 in Combination With Radiotherapy in the Treatment of Locally Advanced Head and Neck Cancer Patients. J Clin Oncol (2004) 22(9):1646–54. doi: 10.1200/JCO.2004.03.089 15117987

[44] Garrido G Tikhomirov IA Rabasa A Yang E Gracia E Iznaga N. Bivalent Binding by Intermediate Affinity of Nimotuzumab: A Contribution to Explain Antibody Clinical Profile. Cancer Biol Ther (2011) 11(4):373–82. doi: 10.4161/cbt.11.4.14097 21150278

[45] Du XX Yu R Wang ZF Du DC Liu QY Wang RM. Outcomes and Prognostic Factors for Patients With Cervical Esophageal Cancer Undergoing Definitive Radiotherapy or Chemoradiotherapy. Bosn J Basic Med Sci (2019) 19(2):186–94. doi: 10.17305/bjbms.2019.3873 PMC6535383 30877837

[46] Badakhshi H Kaul D Zhao KL. Association Between the Inflammatory Biomarker, C-Reactive Protein, and the Response to Radiochemotherapy in Patients With Esophageal Cancer. Mol Clin Oncol (2016) 4(4):643–7. doi: 10.3892/mco.2016.753 PMC4812579 27073683

[47] Huang Y Feng JF Liu JS Chen QX. Prognostic Role of Serum C-Reactive Protein in Esophageal Cancer: A Systematic Review and Meta-Analysis. Ther Clin Risk Manag (2015) 11:89–94. doi: 10.2147/TCRM.S70954 25653533 PMC4309787

[48] Xu S Ramos-Suzarte M Bai X Xu B. Treatment Outcome of Nimotuzumab Plus Chemotherapy in Advanced Cancer Patients: A Single Institute Experience. Oncotarget (2016) 7(22):33391–407. doi: 10.18632/oncotarget.8516 PMC5078104 27050148

[49] Wang C Fu X Cai X Wu X Hu X Fan M. High-Dose Nimotuzumab Improves the Survival Rate of Esophageal Cancer Patients Who Underwent Radiotherapy. Onco Targets Ther (2016) 9:117–22. doi: 10.2147/OTT.S89592 PMC4699509 26766917

[50] Zhao KL Hu XC Wu XH Fu XL Fan M Jiang GL. A Phase I Dose Escalation Study of Nimotuzumab in Combination With Concurrent Chemoradiation for Patients With Locally Advanced Squamous Cell Carcinoma of Esophagus. Invest New Drugs (2012) 30(4):1585–90. doi: 10.1007/s10637-011-9735-0 21901403

